# Age‐regulated cycling metabolites are relevant for behavior

**DOI:** 10.1111/acel.14082

**Published:** 2024-01-11

**Authors:** Jessica E. Schwarz, Arjun Sengupta, Camilo Guevara, Annika F. Barber, Cynthia T. Hsu, Shirley L. Zhang, Aalim Weljie, Amita Sehgal

**Affiliations:** ^1^ Howard Hughes Medical Institute, University of Pennsylvania Philadelphia Pennsylvania USA; ^2^ Chronobiology and Sleep Institute, Perelman School of Medicine, University of Pennsylvania Philadelphia Pennsylvania USA; ^3^ Present address: Waksman Institute and Department of Molecular Biology and Biochemistry, Rutgers The State University of New Jersey New Brunswick New Jersey USA; ^4^ Present address: Department of Cell Biology Emory University School of Medicine Atlanta Georgia USA

**Keywords:** aging, *Drosophila*, lipidomics, metabolomics, neuronal function, sleep

## Abstract

Circadian cycles of sleep:wake and gene expression change with age in all organisms examined. Metabolism is also under robust circadian regulation, but little is known about how metabolic cycles change with age and whether these contribute to the regulation of behavioral cycles. To address this gap, we compared cycling of metabolites in young and old *Drosophila* and found major age‐related variations. A significant model separated the young metabolic profiles by circadian timepoint, but could not be defined for the old metabolic profiles due to the greater variation in this dataset. Of the 159 metabolites measured in fly heads, we found 17 that cycle by JTK analysis in young flies and 17 in aged. Only four metabolites overlapped in the two groups, suggesting that cycling metabolites are distinct in young and old animals. Among our top cyclers exclusive to young flies were components of the pentose phosphate pathway (PPP). As the PPP is important for buffering reactive oxygen species, and overexpression of glucose‐6‐phosphate dehydrogenase (G6PD), a key component of the PPP, was previously shown to extend lifespan in *Drosophila*, we asked if this manipulation also affects sleep:wake cycles. We found that overexpression in circadian clock neurons decreases sleep in association with an increase in cellular calcium and mitochondrial oxidation, suggesting that altering PPP activity affects neuronal activity. Our findings elucidate the importance of metabolic regulation in maintaining patterns of neural activity, and thereby sleep:wake cycles.

## Graphical abstract

The image depicts a metabolomic approach towards identifying a metabolic pathway that regulates behavior in *Drosophila*. Old and young flies were collected at different times of day and heads were subjected to metabolomic analysis to identify cyclically expressed metabolites. The pattern of cycling metabolites was different in young and old animals. Metabolites of the Pentose Phosphate Pathway were found to cycle only in young animals and over‐expression of one of these, G6PD, was previously shown to increase lifespan in *Drosophila*. We show that overexpression of G6PD in circadian neurons also decreases sleep in conjunction with an increase in cellular calcium and ROS. Summary figure was generated using BioRender.

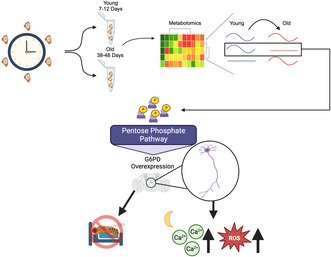

**Abbreviations**
G6PD: glucose‐6‐phosphate dehydrogenase
G6P: glucose‐6‐phosphate
GSH: glutathione
JTK: Jonckheere‐Terpstra‐Kendall
l‐LNv: large ventral lateral neuron
OPLS: Orthogonal projections to latent structures
PPP: pentose phosphate pathway
PDF: pigment dispersing factor
s‐LNv: small ventral lateral neuron
SCN: suprachiasmatic nucleus
DD: total darkness
*w*CS: white CantonS
ZT: zeitgeber time

## 1 INTRODUCTION

A universal hallmark of aging is decreased organismal fitness as the human lifespan exceeds healthspan (Garmany et al., 2021). The circadian clock plays an important role in maintaining synchrony across physiological and behavioral cycles, but circadian regulation changes with age in all organisms examined (Hood & Amir, 2017). For instance, sleep:wake cycles break down, so as humans age, their sleep becomes fragmented (Mander et al., 2017). While the central clock continues to cycle, signaling from the suprachiasmatic nucleus (SCN) degrades with age (Hood & Amir, 2017) seen as desynchrony of firing, changes in GABAergic synapses, as well as changes in the rhythms of cortisol release (Hood & Amir, 2017). Additionally, peripheral circadian rhythms change and may advance in older individuals (Hood & Amir, 2017). In humans, loss of these cycles contributes to other pathologies associated with aging, such as neurodegenerative diseases and memory impairment (Mander et al., 2017).

*Drosophila* exhibit a similar breakdown of circadian rhythms with age. In *Drosophila*, a network of ~150 neurons that express the core molecular clock proteins functions as the central clock (Dubowy & Sehgal, 2017). As in mammals, the transcription translation feedback loop that constitutes the clock mechanism is maintained with age in clock neurons (Luo et al., 2012). However, downstream signals are affected to result in significant arrhythmia and fragmentation of sleep (Koh et al., 2006; Vaccaro et al., 2017). Additionally, peripheral rhythms of gene expression dampen in the body, perhaps because of loss of synchrony across tissues (Luo et al., 2012). Therefore, *Drosophila* are an excellent model to study age‐related changes in circadian physiology.

The circadian clock also plays an important role in maintaining daily cycles in metabolic activity (Marcheva et al., 2013). Organismal fitness likely requires the cycling of cellular metabolism, between anabolic and catabolic (or oxidative and reductive) phases. Metabolic cycles have been found in all species examined and can occur with a periodicity of 4–5 h (in yeast) (Tu et al., 2007), although they are driven to a 24‐hour rhythm in organisms that have circadian clocks. The clock temporally separates metabolic pathways based upon the organism's habitat or behavioral patterns. For instance, specific metabolic pathways may need to occur during the sleep state to support sleep function (Anafi et al., 2019). External enforcement of feeding cycles by time restricted feeding can improve lifespan and healthspan indicating that maintaining such rhythms throughout age is important for organismal fitness (Acosta‐Rodríguez et al., 2021). Surprisingly, despite the relevance of metabolic activity for aging (for instance, many lifespan‐altering manipulations are metabolic in nature), and the profound fitness benefits of cycles in metabolism, little is known about the effects of age on metabolic cycles.

To determine how aging impacts cycles of metabolites, we conducted metabolomic analysis of heads from young and old *Drosophila*. While cycling was generally less robust in old flies, we noted distinct signatures of the young and old cycling metabolome. Components of the PPP were among those whose cycling is compromised by age, and we report here that one of these components affects behavior. Overexpression of G6PD, previously shown to increase lifespan in *Drosophila*, decreases sleep when expressed in circadian clock neurons. This effect of G6PD mimics the decreased sleep phenotype of old flies, but it is also evident in young flies and is accompanied by changes in intracellular calcium and mitochondrial oxidation. Together, these findings identify a cycling metabolic pathway that changes with age and affects sleep behavior.

## 2 RESULTS

### 2.1 Distinct metabolites cycle in young and old fly heads

**FIGURE 1:**
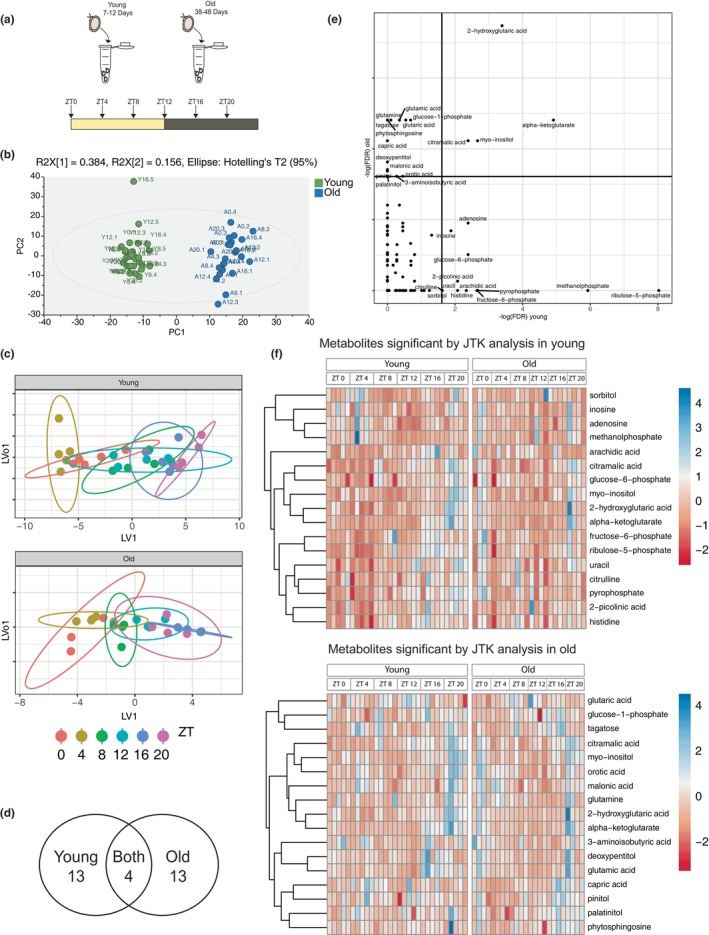
Most of the metabolites cycling in young and old fly heads are distinct. A visual representation of the experimental paradigm for fly head collections at six circadian timepoints (a). Multivariate analysis (OPLS) of the metabolomics data from both young and old flies at six circadian timepoints indicates that major variation in the dataset is age related (b). While no significant time‐based model could be build using the old metabolomic samples, the OPLS scores plot for young flies shows time dependent variation (c). The metabolomics data were analyzed using JTK analysis (BHQ < 0.2) and are presented as a Venn diagram (d). A FDR plot provides visual representation of the significant cyclers in the young (clustered in the bottom right) and significant cyclers in old (clustered in the top right) (e). Heat maps of the data are shown to illustrate that the same number of metabolites is rhythmic in young and old flies, but that most of the rhythmic metabolites are different in each group (f). Data points in (b, c) represent replicates with ~300 fly heads per sample (b, c). For young *N* = 5 and for old *N* = 4 replicates per timepoint. In the OPLS plots, LV1 refers to latent variable, and LV_o_1 refers to orthogonal LV. In the heatmaps, each metabolite in a separate block is individually scaled to unit variance and the mean centered at zero (f).

We aimed to determine whether metabolic cycles change with age. In order to investigate age‐related changes in the circadian metabolome, we conducted metabolomic analyses of young and old *Drosophila* at six different circadian time points throughout the day (Figure 1a). As significant changes in sleep occur by 35 days of age (Vienne et al., 2016), we used flies older than that for our study (age 38–48 days for our old group compared to 7–12 days for our young group). Since we were particularly interested in changes in the brain, we focused on whole head as opposed to whole body metabolomics. Orthogonal projections to latent structures (OPLS) analysis revealed major variation in the dataset based on age, as the samples clustered into two distinct groups, young and old (Figure 1b). Additionally, we were able to generate a significant OPLS model to segregate the young samples by zeitgeber time (ZT) of collection (Figure 1c). However, this analysis failed to identify a significant model for the old samples suggesting that aging leads to a loss of tightly controlled metabolomic cycling based on time of day.

Although the OPLS model was not significant for time‐of‐day differences as a whole in old samples, JTK (Jonckheere–Terpstra–Kendall) analysis (BHQ < 0.2) revealed that similar numbers of metabolites were rhythmic in young and old flies (Figure 1d). We infer that the amplitude of cycling is generally weaker in old flies, but several metabolites still oscillate significantly. However, the majority of rhythmic metabolites were different in each group (Figure 1e). Even among the metabolites that were rhythmic in both young and old flies, many of the species had different expression profiles between ages (Figure 1f). Thus, rhythms of the metabolome change with age.

We also conducted lipidomics analyses on the same samples and found that the cycling of the lipidome is more prominent in old flies. JTK analysis (BHQ < 0.05) revealed that none of the rhythmic lipids overlapped between groups (Figure S1). Triglycerides comprise a large number of the cycling lipids in old flies (22 out of the 53 cycling lipid species we detected). In addition, fatty acids did not show significant cycling in young flies (Figure S1C,D).

### 2.2 Aging does not affect feeding pattern or quantity in *Drosophila*

To determine whether the differences in cycling metabolites between young and old flies are due to differences in nutrient consumption, we monitored feeding behavior. We used the Automated Recording CAFÉ (ARC) assay to look at feeding rhythms under a 12:12 light: dark schedule over 3 days. In short, the ARC assay measures liquid food consumption on an individual fly basis by tracking the distance traveled by a dye band at the top of the liquid food as the fly eats out of the capillary. We found no significant differences in feeding pattern or total nutrients consumed between young and old flies in this assay (Figure S2). Therefore, the differences in rhythmic metabolites between groups are not due to changes in feeding.

### 2.3 Metabolites of the pentose phosphate pathway lose rhythmicity with age

**FIGURE 2:**
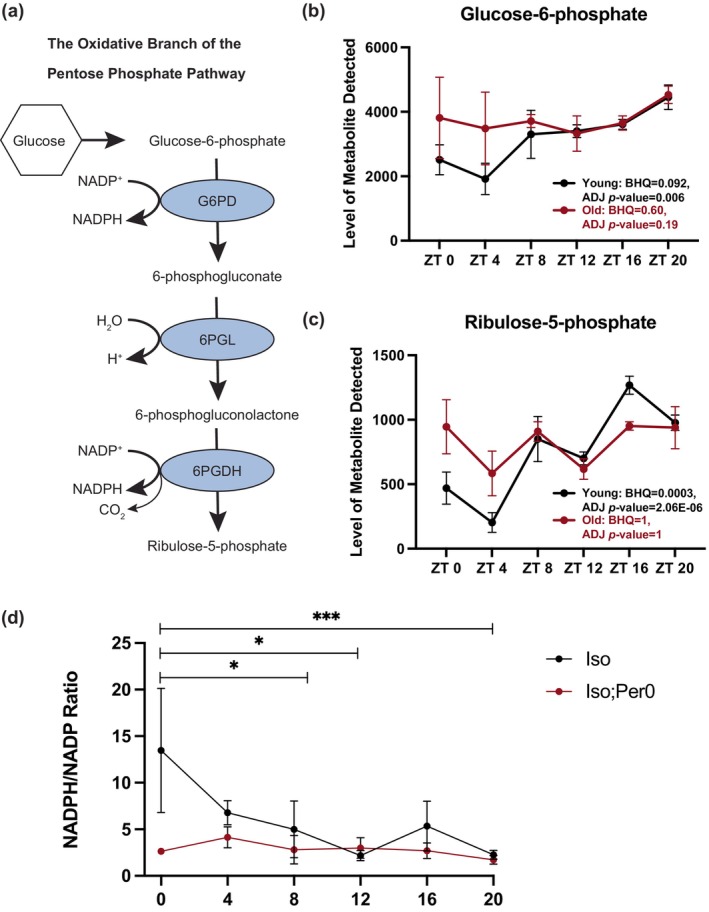
Metabolites in the pentose phosphate pathway lose rhythmicity with age. A schematic of the oxidative branch of the pentose phosphate pathway (PPP) is shown to illustrate the location of Glucose‐6‐Phosphate and Ribulose‐5‐phosphate in the pathway (a). Metabolite expression levels (reported as normalized mTIC averaged peak heights) of two of the most significant cyclers identified by JTK analysis in young flies in Figure 1, are glucose‐6‐phosphate (b) and ribulose‐6‐phosphate (c), are graphed over six circadian timepoints. *N* = 4 for the old and *N* = 5 for the young per timepoint. Data points represent replicates with ~300 fly heads per sample. Error bars are SEM. Activity of glucose‐6‐phosphate dehydrogenase, the rate‐limiting enzyme of the oxidative branch of the PPP, changes during the day and is dependent on the circadian clock (d). NADPH/NADP+ ratios were measured in heads from male and female wild‐type flies (Iso31) and circadian mutants (Iso31, Per0). *N* = 3–6 per timepoint where *N* represents a replicate of ~200 heads. Multiple comparisons were performed among ZT times and corrected with a Bonferroni correction. Error bars are SEM. *p < 0.05, ***p < 0.001.

Since food consumption was not a contributing factor to changes in the diurnal metabolome with age, we sought to identify the endogenous biochemical pathways that exhibit age‐dependent changes in cycling. Scanning the data for metabolites that are rhythmic in young flies but not in old, we identified components of the pentose phosphate pathway (PPP) (Figure 2a). Two of the most significant cyclers in young flies were glucose‐6‐phosphate (young BHQ 0.092, old BHQ: 0.60) and ribulouse‐5‐phosphate (Young BHQ: 0.0003, Old BHQ: 1) (Figure 2b,c), and neither of these showed significant cycling in old flies. Both species are essential components of the oxidative stage of the PPP. Additionally, fructose‐6‐phosphate also cycles in young flies, but not old (Young BHQ: 0.071, Old BHQ: 1) and is a metabolite linked to the PPP further emphasizing the significance of this pathway in our findings.

In the PPP, the rate‐limiting step is conversion of glucose‐6‐phosphate (G6P) to 6‐phosphogluconate, with the concomitant reduction of NADP to NADPH, by the enzyme G6P dehydrogenase (G6PD) (Figure 2a). To more specifically address cycling within the PPP, we measured the NADPH/NADP^+^ ratio in fly heads throughout the day. In addition to heads from wild‐type ISO31 flies, we included circadian mutants *per* ^*0*^ in an Iso^31^ background, which allowed us to test for circadian control. Using a mixed model analysis, we evaluated differences between groups and between ZT times (Figure 2d). We found significant differences in the control group comparing ZT0 and ZT8 (*p*‐value = 0.0405); ZT0 and ZT12 (*p*‐value = 0.0256); and ZT0 and ZT20 (*p*‐value = 0.0008). We did not find significant differences between ZT times in the *per* ^*0*^ flies, suggesting that G6PD activity changes during the day and depends on the circadian clock (Figure 2d). A high NADPH/NADP ratio at ZT0 and declining steadily after that through the middle of the night is consistent with the data in Figure 2b showing that young flies have lower levels of G6P, and thereby higher G6PD enzyme activity, at ZT0 and ZT4. Old flies have high levels of G6P throughout the day, suggesting reduced activity of G6PD.

### 2.4 G6PD overexpression and knockdown in *PDF*+ clock neurons decreases sleep

Our finding that PPP metabolites change with age is consistent with the critical role they play in redox maintenance via production of NADPH and glutathione (GSH). Interestingly, naturally occurring variants of the G6PD gene *Zw* are known to increase G6PD activity and also promote longevity (Luckinbill et al., 1990). Moreover, overexpression of G6PD using a variety of different drivers leads to lifespan extension in *Drosophila* and increased ability to survive oxidative stress (Legan et al., 2008). G6PD overexpression has also been used to ameliorate the effects of neurodegenerative disease in *Drosophila* (Besson et al., 2015). The relevance of G6PD overexpression for aging led us to ask whether it affects behavior that changes with age, namely sleep:wake cycles.

**FIGURE 3:**
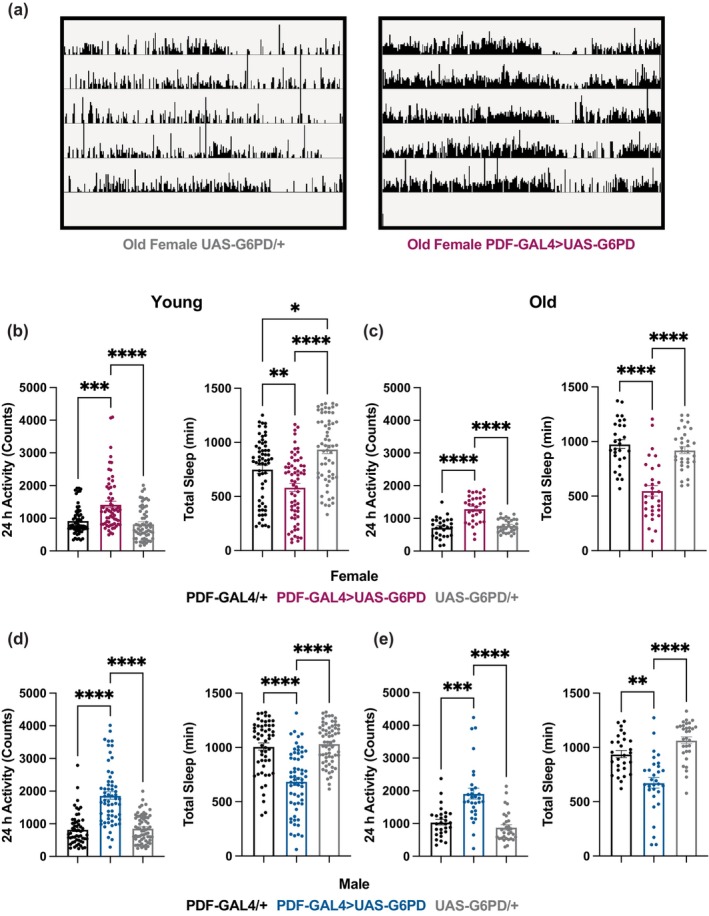
G6PD Overexpression in PDF^+^ clock neurons increases locomotor activity. Transgenic flies overexpressing G6PD in clock neurons were assayed for overall activity levels and total sleep for 5 days in DD (a). Representative actograms showing increased activity in old female flies with G6PD overexpression compared to control over 5 days in DD. Data for each female (b, c) and male (d, e) young (b, d) and old (c, e) exhibited an increase in overall locomotor activity when G6PD was overexpressed in PDF^+^ neurons. Circles are individual fly data points, and summary statistics are displayed as mean + SEM. Means are compared by Tukey's test following one‐way ANOVA (c) or Kruskal–Wallis test followed by Dunn's test (b, d, and e). *N* = 28–63 **p < 0.01, ***p < 0.001, ****p < 0.0001.

Circadian regulation of sleep:wake behavior depends upon a molecular clock that localizes to 150 central clock neurons in the *Drosophila* brain (Dubowy & Sehgal, 2017). Of these, perhaps the most important group are the pigment dispersing factor (PDF^+^) neurons, which are called morning cells because they maintain the morning activity peak of locomotor activity in *Drosophila* (Grima et al., 2004), and are comprised of large (l‐LNvs) and small (s‐LNvs) ventral lateral neurons. PDF^+^ neuronal function is disrupted with aging, in association with disruptions in behavioral rhythms. With aging, PDF expression decreases in the s‐LNVs and their dorsal projections; and, overexpression of PDF in aged flies can rescue locomotor rhythm deficits (Vaccaro et al., 2017). Additionally, the daily remodeling of PDF^+^ axon terminals is attenuated with age (Nguyen et al., 2022). Thus, aging impacts both G6PD and PDF^+^ neurons, leading us to ask whether overexpression of G6PD in PDF^+^ clock neurons impacts circadian rhythms of locomotor activity and sleep. We monitored locomotor activity in total darkness (DD) to look at circadian behavior in old and young flies overexpressing G6PD in PDF^+^ neurons. We only considered behavioral data significant when the experimental genotype was significantly different from both controls. G6PD overexpression did not improve rhythms, but rather weakened rhythms in old female flies (Figure S3). The most striking phenotype was a dramatic increase in activity counts across all groups (Figure 3a–e, left). Based on this finding, we were curious as to whether this increase in activity counts corresponded to a decrease in sleep. We binned the DD data using the 5‐minute definition of sleep in *Drosophila* and found that in both young and old *Drosophila* G6PD overexpression in PDF^+^ neurons significantly decreased sleep (Figure 3b–e, right).

**FIGURE 4:**
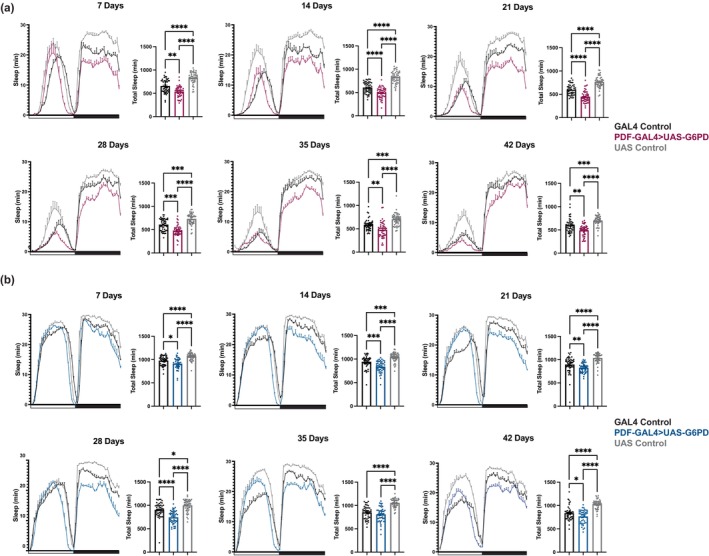
G6PD Overexpression in PDF^+^ clock neurons decreases sleep. G6PD overexpression in PDF^+^ neurons decreases total sleep (a, b). Average baseline sleep profiles of female (a) and male (b) transgenic flies with the indicated ages and genotypes are shown to illustrate the effects of G6PD on total sleep. Graphs of total sleep for each age group to the right of each sleep profile are shown to demonstrate the significant effects of G6PD overexpression. White bar on *X* axis = lights on (day); Black bar on *X* axis = lights off (night). The data for individual flies are presented with the mean +/− SEM for the group. *N* = 41–48 per group. Means are compared by Kruskal–Walis except female 21 days and 14 days as well as male 42 days and 35 days which were all compared by one‐way ANOVA *p < 0.05, **p < 0.01, ***p < 0.001, ****p < 0.0001.

In order to more closely monitor age‐associated changes in sleep:wake and activity, and determine how these are affected by G6PD overexpression, we monitored flies at 7, 14, 21, 28, 35, and 42 days of age. Each genetic control and experimental group showed distinct signs of behavioral aging from 7 to 42 days, which typically manifested as a decrease in total sleep and waking activity (Figure S4), consistent with previous findings (Vienne et al., 2016). Female flies overexpressing G6PD did not show a decrease in sleep on Day 42 relative to Day 7, likely because sleep in these flies was already quite low; however, they did show decreased waking activity with age. G6PD overexpression was found to decrease total sleep, relative to controls, across all age groups and both sexes (Figure 4). In particular, we noticed a striking decrease in sleep at night (Figure 4). Additionally, G6PD overexpression increased waking activity across all age groups in the male flies and at 28 days and 42 days in female flies (Figure S5). Thus, while effects of G6PD overexpression on sleep are largely age‐independent, the improvement of waking activity in older females suggests an interaction with age.

Given that G6PD overexpression decreased sleep, we were interested to determine whether knocking down G6PD also had an effect on sleep. Since G6PD overexpression had similar effects on behavior in old and young flies, we focused subsequent analysis on young flies. We used two different RNAi lines (VDRC 101507 and 3337) previously shown to decrease G6PD expression (Xie et al., 2013), and found that both RNAi lines decreased total sleep in female and male flies (Figure S6). We infer that tight regulation of PPP activity of the PPP is important for maintaining normal sleep patterns.

### 2.5 G6PD overexpression in PDF^+^ clock neurons increases nighttime calcium levels and oxidation

**FIGURE 5:**
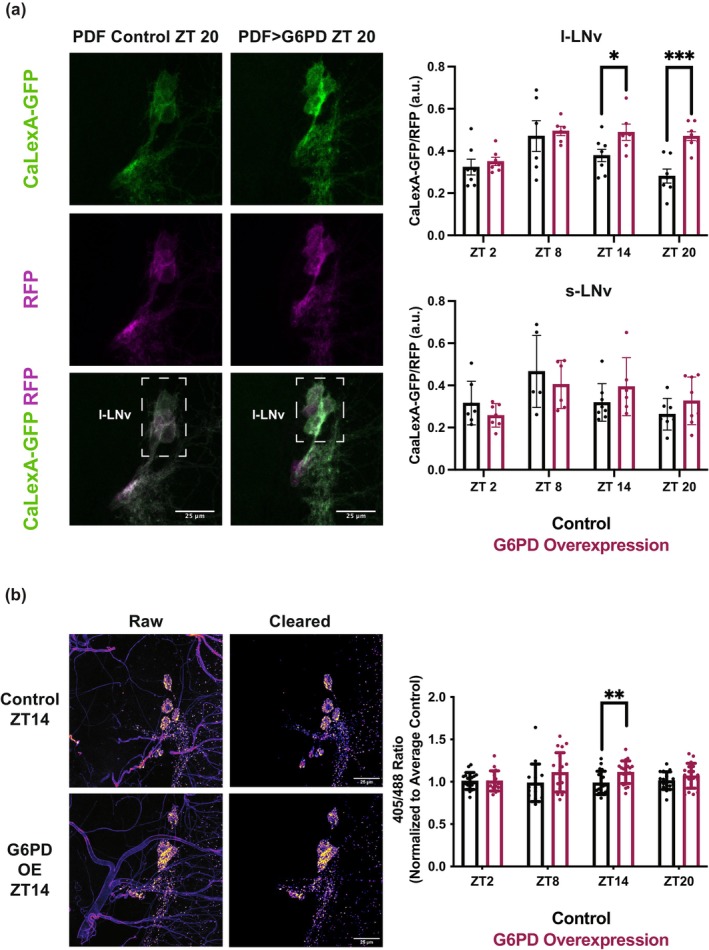
G6PD overexpression in PDF^+^ neurons increases nighttime oxidation and calcium in PDF^+^ neurons. (a) Representative images are shown to demonstrate the increased calcium via CaLexA‐GFP signal normalized by RFP in l‐LNv PDF+ neurons at ZT 20 in control vs. and G6PD overexpression flies. Quantitative analysis of these images was performed to determine the Ca^2+^ levels in the neurons, and the data for each timepoint are presented with the mean +/− SEM. Note that Ca^2+^ levels are significantly increased in G6PD overexpression flies at ZT14 and ZT20 compared to control. *N* = 6–8 per group. (b) Representative images of PDF+ neurons show increased oxidation reduction ratio (405 nm/488 nm signal) via mitoRo‐GFP signal in l‐LNv PDF+ neurons at ZT 14 in G6PD overexpression vs. control flies. Images are displayed with “fire” pseudocolor of mitoRo‐GFP signal (blue/purple = low intensity and yellow/white = high intensity). Left most images are raw data while the right image shows the signal measured using custom macro to eliminate background. Quantitative analysis of these images was performed to determine the oxidation levels in the neurons, and the data for timepoint are presented with the mean +/− SEM. Note the significant increase at ZT14 in G6PD overexpression flies vs. control. *N* = 15–19 per group. Data points are an average of individual cells (a) or an average of signal (b) per individual fly, and summary statistics are displayed as mean +/− SEM. Means compared by unpaired *t*‐test (a) or Welch's *t*‐test (b). *p < 0.05, **p < 0.01, ***p < 0.001.

PDF^+^ cells, especially the l‐LNvs, promote locomotor activity and reduce sleep, suggesting that the decreased sleep produced by G6PD overexpression results from increased activity of these cells. To determine whether this was the case, we examined effects of G6PD overexpression on calcium levels, using the genetically encoded, transcriptionally activated calcium reporter (CaLexA) where GFP signal is a proxy for calcium level in the cell (Masuyama et al., 2012). Because we measured CaLexA signal in individual cells, we were able to differentiate the s‐LNv and l‐LNv subpopulations. The results showed that nighttime calcium, ZT14 and ZT20, was higher compared to control flies in the l‐LNvs, but not the s‐LNvs (Figure 5a). This is consistent with previous findings that l‐LNvs in particular are arousal‐promoting cells (Sheeba et al., 2008). Also, in examining the sleep profiles of the G6PD‐overexpressing flies (Figure 4), we note that the timing of the increase in calcium corresponds to the time at which G6PD‐overexpressing flies are active, while their controls are sleeping.

Given that the PPP plays an important role in redox buffering, we tested whether the oxidation state of PDF^+^ neurons was also affected by G6PD overexpression in a time‐of‐day dependent manner. To measure redox state, we used a genetically encoded mitochondrial glutathione redox sensor, mito‐roGFP2‐Grx1 (Albrecht et al., 2011), whose fluorescence intensity ratio 405 nm/488 nm indicates redox state. We collected flies at four different circadian timepoints (ZT2, ZT8, ZT14, and ZT20) and assayed fluorescence of the sensor at 405 and 488 nm in PDF^+^ cells. We found that relative to control flies G6PD overexpression resulted in a higher 405 nm/488 nm ratio at ZT14 (Figure 5b), indicating a more oxidized state. While the peak change doesn't align perfectly with the CaLexA peak, the CaLexA signal is a summation of calcium activity over the last ~4 h, so these results do align. Thus, the decrease in sleep, increase in calcium, and increase in mitochondrial oxidation all occur at the same time of day.

### 2.6 G6PD overexpression in some, but not all, sleep‐relevant neurons decreases sleep

**FIGURE 6:**
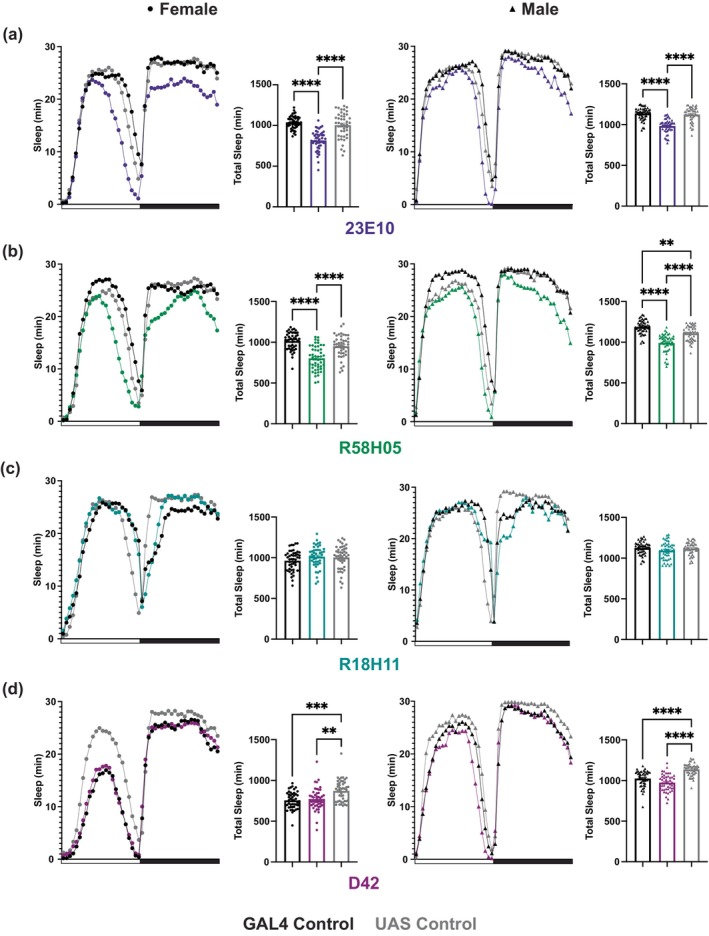
G6PD overexpression in some sleep relevant, but not all neural populations decrease sleep. Sleep studies were performed on young transgenic flies to determine the average baseline sleep profiles and total sleep measured when G6PD is overexpressed in four different neural populations: 23E10 (a), R58H05 (b), R18H11 (c), and D42 (d). Average baseline sleep profiles are shown for females (left) and males (right), and the data from individual flies are graphed with mean +/− SEM of the group. Note that overexpressing G6PD with 23E10‐GAL4 and R58H05‐GAL4 significantly decreases sleep vs. both GAL4 and UAS controls. *N* = 43–48 per group. X‐axis bars indicate light conditions. White: lights on (day); Black: lights off (night). Circles are individual fly data points, and summary statistics are displayed as mean +/− SEM. Means compared by one‐way ANOVA (a‐Female, b‐Female, c‐Female, c‐Male) or Kruskal–Wallis (a‐Male, b‐Male, d‐Female, d‐Male). *p < 0.05, **p < 0.01, ***p < 0.001, ****p < 0.0001.

While we originally focused on PDF^+^ neurons because of their fundamental role in maintaining circadian rhythms of sleep:wake, we were also interested in testing whether G6PD overexpression in other sleep and circadian relevant neurons affects sleep in *Drosophila*. G6PD overexpression in both the dorsal fan shaped body (23E10‐GAL4) and ellipsoid body (R58H05‐GAL4) neurons led to decreased sleep (Figure 6a,b). However, overexpression of G6PD in the DN1 neurons, which are clock neurons implicated in sleep regulation (Guo et al., 2016), does not affect sleep (Figure 6c). Thus, G6PD overexpression decreases sleep in some, but not all sleep/circadian related neurons. Additionally, we were curious if the previously reported extension in lifespan with G6PD overexpression (Legan et al., 2008) driven by D42‐GAL4 could be related to a decrease in sleep. The results showed that D42 > G6PD does not alter total sleep (Figure 6d), so changes in sleep likely do not contribute to effects on lifespan. Taken together, these findings demonstrate that the link between G6PD overexpression and sleep amount is not exclusive to PDF^+^ neurons, but also is not a universal phenomenon in all neuronal subsets.

## 3 DISCUSSION

While transcript cycling has been studied extensively by circadian researchers, including in the context of age (Kuintzle et al., 2017), effects of aging on metabolite cycling are not known. We demonstrate here that in *Drosophila* heads there is a dramatic shift in metabolite cycling with age such that only four cyclic metabolites and no cyclic lipid species overlap between old and young. We identified the pentose phosphate pathway as one of the significant pathways enriched in terms of metabolite cycling in young flies. We also find that overexpressing glucose‐6‐phosphate, the rate‐limiting enzyme in the oxidative branch of the PPP, in PDF^+^ neurons decreases sleep. Additionally, G6PD overexpression leads to increased calcium activity and oxidation in PDF^+^ neurons at the same time of day as the decreased sleep, providing cellular correlates for the observed behavioral phenotype. Thus, modulating the activity of a metabolic pathway affected by aging can result in behavioral changes and altered cellular physiology.

We previously demonstrated that oscillations of clock genes, as assayed in whole heads or bodies, dampen with age in *Drosophila* (Luo et al., 2012). Therefore, we hypothesized that metabolic rhythms in heads would dampen or disappear with age. However, we observed an equal number of cycling metabolites in both young and old flies, although there was a change in the metabolites that were rhythmic in old flies. Furthermore, there was a strong increase in cycling of lipids with age, with no overlap of cycling lipids between young and old. The significantly larger number of cycling lipids in old flies suggests that the cycling lipidome not only changes, but also becomes more prominent with age. We found that the feeding patterns of the young and old flies are identical. Thus, the changes we observed in cycling metabolites and lipids were not due to the amount of nutrients consumed or the timing of feeding. While this distinct switch in the rhythmic metabolome and lipidome was not what we originally anticipated, the idea of circadian reorganization with age has been demonstrated before with transcriptomics. In the mouse liver, there are distinct populations of genes that either gain or lose rhythm with age (Sato et al., 2017). Additionally, in the *Drosophila* head some transcripts lose cycling with aging while others gain rhythmicity (Kuintzle et al., 2017). Ours are the first data to demonstrate that the circadian metabolome can change under different conditions. Identifying pathways that cycle in either young or old flies provides insight into potential targets to ameliorate the detrimental effects of aging.

We identified two metabolites in the pentose phosphate pathway that lose rhythmicity with age. Robust cycling of this pathway in young flies is supported by our finding that, in addition to the two cycling metabolites identified through metabolomics, the NADPH/NADP+ ratio changes over a daily cycle in a clock dependent fashion. Also, glutathione which is reduced by NADPH was previously shown to be circadian regulated (Beaver et al., 2012). Given its role in redox buffering in the cell, it is perhaps not surprising that the PPP is implicated in aging. As mentioned above, increasing G6PD activity increases longevity in Drosophila (Legan et al., 2008; Luckinbill et al., 1990) and mitigates the effects of neurodegenerative disease (Jeng et al., 2013). In mice, G6PD overexpression increases NADPH levels and decreases oxidative damage in older mice to lead to increased healthspan (Nóbrega‐Pereira et al., 2016). It has also been shown that modulating PPP activity can shift the circadian clock (Rey et al., 2016). Thus, loss of PPP cycling with age is of particular interest. While G6PD overexpression had limited age‐specific effects in the behaviors we examined here, it is likely that this manipulation or loss of PPP cycling impact aging physiology in other ways.

We find that overexpressing G6PD in PDF^+^ clock neurons affects sleep, nighttime neuronal calcium, and nighttime mitochondrial oxidation. PDF^+^ clock neurons are known to promote wake. Specifically, constitutive activation of LNvs, in particular the l‐LNvs, decreases nighttime sleep and increases mean activity counts during both day and night (Sheeba et al., 2008). We find a very similar change in sleep with G6PD overexpression in PDF^+^ neurons, suggesting that G6PD activates these neurons. This was supported by the increase in nighttime calcium in specifically the l‐LNvs with G6PD overexpression. We propose that increasing G6PD activity leads to increased neuronal activity, which in the case of l‐LNvs, leads to a decrease in sleep. Whether the increased nighttime oxidation is a cause or a consequence of increased neuronal activity is unclear. However, given that G6PD typically enhances NADPH/GSH production, and overexpression decreased oxidation in mice, the mitochondrial oxidation observed here likely results from increased neuronal activity and not directly from the extra G6PD. On the other hand, it is possible that dysregulated G6PD has adverse effects on the redox balance in some cells. For instance, even the RNAi knockdown of G6PD in PDF^+^ neurons lead to a decrease in sleep demonstrating that disruption of G6PD activity in either direction alters sleep behavior. Notably, increased oxidation globally or in sleep‐promoting cells has been shown to increase sleep, but could have different effects in wake‐promoting neurons.

While we do not know how increasing G6PD activity alters neuronal calcium, we have a few potential hypotheses. Downregulation of the GABA_A_ receptor subunit *Rdl* in the PDF^+^ neurons leads to decreased sleep (Parisky et al., 2008), that is, a similar phenotype to that produced by G6PD overexpression. Given that GABA signaling is modulated by redox levels, in particular by ROS (Beltrán González et al., 2014, 2020), it is tempting to speculate that GABA receptor expression is altered by increased G6PD activity. Another possibility is that G6PD directly alters the activity of calcium channels. In mammalian cultures, G6PD is known to bind to calcium channel Ca_V_1.2 to increase intracellular calcium directly (Gupte et al., 2020). These hypotheses suggest additional questions for future investigation.

We find that while overexpressing G6PD in the dFB and EB also decreases sleep, overexpressing G6PD in the DN1 neurons does not affect sleep amount. Thus, effects of G6PD on sleep are not universal across sleep‐regulating neurons. Given that GABA signaling is implicated in effects of the dFB and the EB (Kempf et al., 2019; Kim et al., 2020; Ni et al., 2019), these findings would be consistent with the idea that G6PD interacts with GABA signaling to modulate sleep. The negative result from the DN1 neurons is particularly of note because they are both circadian and sleep regulating. In addition, the number of DN1 neurons is larger than the number of PDF^+^ neurons, so the effect on sleep is not dependent on the spatial extent of expression. Further investigation of the neurotransmitters, neuropeptides, and channels expressed by the different subsets might help elucidate the mechanism by which altering G6PD activity leads to decreased sleep.

The link we demonstrate here between the enzymatic activity of a specific metabolic pathway and neuronal activity has implications well beyond the current study. Although metabolic determinants of neural function are now being appreciated, there is still much to learn about them. In addition to the pathway we focused on here, we expect that other metabolic pathways implicated by our data provide potential targets for behavioral modulation, perhaps with respect to aging.

## 4 MATERIALS AND METHODS

### 4.1 Fly husbandry

Flies were raised on a cornmeal‐molasses diet at 25°C. Metabolomics/lipidomics/feeding assays were conducted with white CantonS (*w*CS) flies (gift from L. Griffith lab). For sleep/circadian experiments, Gal4/UAS lines were tested as heterozygotes crossed to *w*CS to avoid testing behavior with balancers. UAS‐G6PD9g was a gift from W. Orr (Legan et al., 2008). PDF‐GAL4 was a gift from J. Hall. The per01 flies (Flybase: FBal0013649) originally from (Konopka & Benzer, 1971) were backcrossed to Iso^31^. The following lines were acquired from Blooming Stock Center: 23E10‐GAL4 (#49032) backcrossed to Iso^31^, R18H11‐GAL4 (#48832), R58H05‐GAL4 (#39198) backcrossed to Iso^31^, D42‐GAL4 (#8816) backcrossed to Iso^31^, UAS‐mito‐roGFP2‐Grx1 (#67664). The mito‐roGFP; UAS‐mito‐mCherry fly line was created by P. Haynes (A.S. laboratory). The CaLexA fly was a gift from J. W. Wang (Masuyama et al., 2012). The CaLexA fly with UAS‐CD8‐RFP was created by A. N. King (A.S. laboratory) (Schwarz et al., 2021).

### 4.2 Metabolomics/lipidomics

Collected and sorted *w*CS flies into bottles of 150. Flies were flipped every 2–4 days to maintain flies throughout the course of the aging process. Young flies were 7–12 days old and old flies were 38–48 days old at collection. All flies were anesthetized with CO2, collected, frozen with dry ice. Flies were vortexed and sieved to collect fly heads and immediately stored at −80°C in groups of ~300 heads per sample. Samples were sent to UC Davis West Coast Metabolomics Core (WCMC) for metabolomic and lipidomic analysis. WCMC performed primary metabolism by GCTOF MS using a Restek corporation Rtx‐5Sil MS (30 m length × 0.25 mm internal diameter with 0.25 μm film made of 95% dimethyl/5%diphenylpolysiloxane) column. A more detailed protocol was reported in previous literature (Fiehn et al., 2008). ChromaTOF vs. 2.32 was used for metabolomics data preprocessing without smoothing, 3 s peak width, baseline subtraction just above the noise level, and automatic mass spectral deconvolution and peak detection at signal/noise levels of 5:1 throughout the chromatogram. Apex masses are reported for use in the BinBase algorithm. For lipidomics by CSH‐ESI QTOF MS/MS, lipids were extracted using the Matyash protocol (Matyash et al., 2008). Mass spectrometry parameters are used as follows: for positively charged lipids such as PC, lysoPC, PE, PS, an Agilent 6530 QTOF mass spectrometer is used with resolution *R* = 10,000 while negatively charged lipids such as free fatty acids and phosphatidylinositols were also analyzed using an Agilent 6530 QTOF mass spectrometer with resolution *R* = 10,000. Data were analyzed using MS‐Dial v 3.52 for untargeted features, target metabolite detection, peak alignment, and filtering. LipidBlast was used for lipid annotations. For both metabolomics and lipidomics, raw data were normalized and are presented as mTIC values. Circadian statistical analysis was performed in R using JTK_CYCLEv3.1 (Hughes et al., 2010).

### 4.3 Multivariate data analysis of metabolomics data

Principal component analysis (PCA) and orthogonal partial least square regression: All multivariate analysis were performed using Simca‐P 17.0 software (Sartorius AG, Germany). Datasets were imported into the software, followed by unit variance scaling and mean centering. Variables were transformed as necessary. PCA model was initially built to check for any obvious outlier and/or trend in the data and general data quality check. No noticeable outlier samples were observed. OPLS regression was carried out separately for young and old datasets using ZT values as the dependent variables and the metabolomic dataset as the independent variable. OPLS model was constructed using 7‐fold cross validation. The robustness of the model was judged using Q2(cum) and CV‐ANOVA p statistic.

### 4.4 Automated recording Café assay

Single‐fly circadian feeding was assessed using the Automated Recording CAFE assay (Murphy et al., 2017), with modifications as described by (Barber et al., 2021). In brief, 5–7 days old male flies were entrained to a 12:12 LD cycle for at least 3 days prior to assay loading and maintained in LD throughout the assay. Flies were individually housed in plastic chambers on medium consisting of 2% agar with a 5 μL calibrated capillary containing a 2.5% sucrose, 2.5% yeast extract liquid diet layered with an infrared‐absorbent dye inserted into each chamber from above. Due to evaporation, capillaries were changed daily throughout the duration of the assay during the morning activity peak. Data were acquired using web cameras (Microsoft LifeCam) modified with an infrared pass filter, through the JavaGrinders‐ARC software (http://javagrinders‐arc.blogspot.com) and processed using the Noah python script (https://github.com/HungryFly/flyARC) to generate single‐fly feeding data in one‐minute bins. Food consumption per 24‐hour day was scored for rhythmicity by JTK_Cycle (Hughes et al., 2010); mean total food intake was compared between ages by Mann–Whitney test.

### 4.5 Circadian/sleep analysis

Flies were individually housed in glass vials with 2% agar 5% sucrose at 25C. Activity was monitored using the Trikinetics (https://trikinetics.com/) *Drosophila* Activity Monitoring (DAM) system. For circadian assays, activity was monitored in total darkness. Old flies were loaded at 35–38 days, and young flies were loaded at 5–10 days. Data were analyzed for 5 days. To identify and eliminate artifacts in the circadian data, a custom Matlab script was written to pool non‐zero activity counts per minute for all experiments. Outliers were identified as values at least six standard deviations above the mean and were replaced with the average between activity counts in the minute preceding and the minute following the bin in which the outlier was found. 24 h FFT and total activity were calculated in Clocklab (https://actimetrics.com/products/clocklab/). Sleep was calculated by a custom Matlab script (Hsu et al., 2021) using the 5‐minute definition of sleep. Sleep analysis of the circadian data was calculated using the same 5 days of analysis as the circadian analysis. All other sleep experiments were run in 12 light:12 dark (LD) cycle. LD sleep was calculated using the first four full days in the assay. All sleep experiments used flies loaded ~7 days old except for the multibeam experiment where approximate loading ages are specified. Behavior experiments were conducted in 2–4 independent runs. All behavioral assays were conducted in single beam monitors except for the characterization G6PD overexpression across six age groups which was measured using updated multibeam monitors (Trikinetics). Changes in behavior were only considered significant if experimental condition was significantly different from both controls (GAL4 and UAS). Code for filtering circadian data and analyzing sleep behavior can be found at https://github.com/cthsu86/damSleepConverter (saveCompositeDataToMat.m and saveCompositeDataToMat_withRewrite.m were written specifically for this manuscript).

### 4.6 CaLexA

After at least 5 days of proper entrainment, young flies age 7–10 days were dissected at ZT 2, 8, 14, and 20 in PBST (PBS + 0.3%TritonX‐100). Brains were fixed for at least 20 min in 4% paraformaldehyde (PFA) at room temperature. Blocking solution, 5% v/v NGST (Normal Goat Serum diluted in PBST), was applied for an hour at 4°C on a nutator. Brains were left in the primary antibodies, Rat anti‐RFP 5F8 Chromotek (1:1000) and Rabbit anti‐GFP A11122 Invitrogen (1:1000), diluted in PBST at 4°C overnight. After ~30 min of washes in PBST on a nutator, brains were left in secondary antibodies, Goat anti‐Rabbit IgG (H + L) Cross‐Adsorbed Secondary Antibody Alexa Fluor 488 A‐11008 Fisher (1:1000) and Goat anti‐Rat IgG (H + L) Cross‐Adsorbed Secondary Antibody Alexa Fluor 594 A‐11007 Fisher (1:1000), on a nutator at 4°C overnight. After ~30 min of washes in PBST on a nutator, PBST was replaced with 50% glycerol/50% PBST. Brains were mounted in Vectashield media (Vector Laboratories Inc.). Eight‐bit images were acquired using a Leica TCS SP5 laser scanning confocal microscope with a 40×/1.3 numerical aperture (NA) 0.49 μm z‐step size. To analyze the images, fluorescence intensity measurement was performed in Fiji, a distribution of ImageJ software (Schindelin et al., 2012). Individual cells were identified in the 594 channel by hand drawn ROIs on the slice where the cell was most present. Approximately equal size ROIs were draw on the background of each slice where a cell was identified. CaLexA signal in each cell was defined as (GFP signal/Background signal on that slice)/(RFP signal/Background signal on that slice). Each data point represents the average A.U. (arbitrary units from ratio normalization) of all l‐LNvs or s‐LNvs identified in a single brain.

### 4.7 RoGFP

After at least 5 days of proper entrainment, young flies age 5–12 days were dissected in saline (Wilson et al., 2004) at ZT 2, 8, 14, and 20. Dissected brains were put in 20 mM N‐Ethylmaleimide (NEM) for 10 min at room temperature. Brains were then rinsed with saline and fixed with 2% PFA overnight at 4°C. The next day brains were washed with PBST (PBS + 0.3%TritonX‐100) and put in 50% glycerol/50% PBST. Brains were mounted in Vectashield media (Vector Laboratories Inc.). Eight‐bit images were acquired using a Leica Stellars 8 laser scanning confocal microscope with a 20×/0.7 numerical aperture (NA) 0.49 μm z‐step size. Images were analyzed using a custom ImageJ macro to threshold the image to select the mitochondria and exclude background/trachea signal. The 405/488 ratio for each brain was normalized to the average ratio of a control brain (across all timepoints) in that run.

## AUTHOR CONTRIBUTIONS

Conceptualization, J.E.S. and A.S.; Methodology, J.E.S, Ar. S., C.G., A.F.B., C.T.H., S.L.Z., A.W., and A.S.; Formal Analysis, J.E.S., Ar. S., C.G., A.F.B., C.T.H., and S.L.Z; Investigation, J.E.S, Ar. S., C.G., A.F.B., C.T.H., and S.L.Z; Writing—Original Draft, J.E.S. and A.S.; Writing—Review & Editing, J.E.S., Ar. S., C.G., A.F.B., C.T.H., S.L.Z, A.W., and A.S.; Visualization, J.E.S., Ar. S., C.G., and A.F.B.; Supervision, A.S.

## FUNDING INFORMATION

This study was supported by the National insitutes of Health (NIH) grants DK120757 (A.W. and A.S.), NS048471 (A.S.), Diversity Supplement NS048471 (J.E.S.), and T32‐NS105607 (J.E.S.). Additional funding for this study came from the Howard Hughes Medical Institute (A.S.) and the Howard Hughes Medical Institute James H. Gilliam Fellowship for Advanced Study program (J.E.S.).

## CONFLICT OF INTEREST STATEMENT

The authors have no conflict of interest to declare.

## STATISTICAL ANALYSIS

All statistics were run in Prism 10.0.0. The Shapiro–Wilk test for normality determined which statistical tests to run. If any groups in a panel did not pass, nonparametric tests were used (Mann–Whitney or Kruskal–Wallis with Dunn's multiple comparisons test). If all groups were normal, a *t*‐test or one‐way ANOVA with Tukey's test was used.

## Supporting information


Figures S1–S6

## Data Availability

All data are available upon request (amita@pennmedicine.upenn.edu). Metabolomics and lipidomics data can be found at PRJNA868356 (NCBI BioProject).
