# Supplementary material for: Age‐regulated cycling metabolites are relevant for behavior
Source: Aging Cell. 2024 Jan 11;23(4):e14082. doi: 10.1111/acel.14082 (PMC11019118; doi:10.1111/acel.14082)
Supplement: Supplementary file 1 — Figures S1–S6 [file ACEL-23-e14082-s001.docx]

**
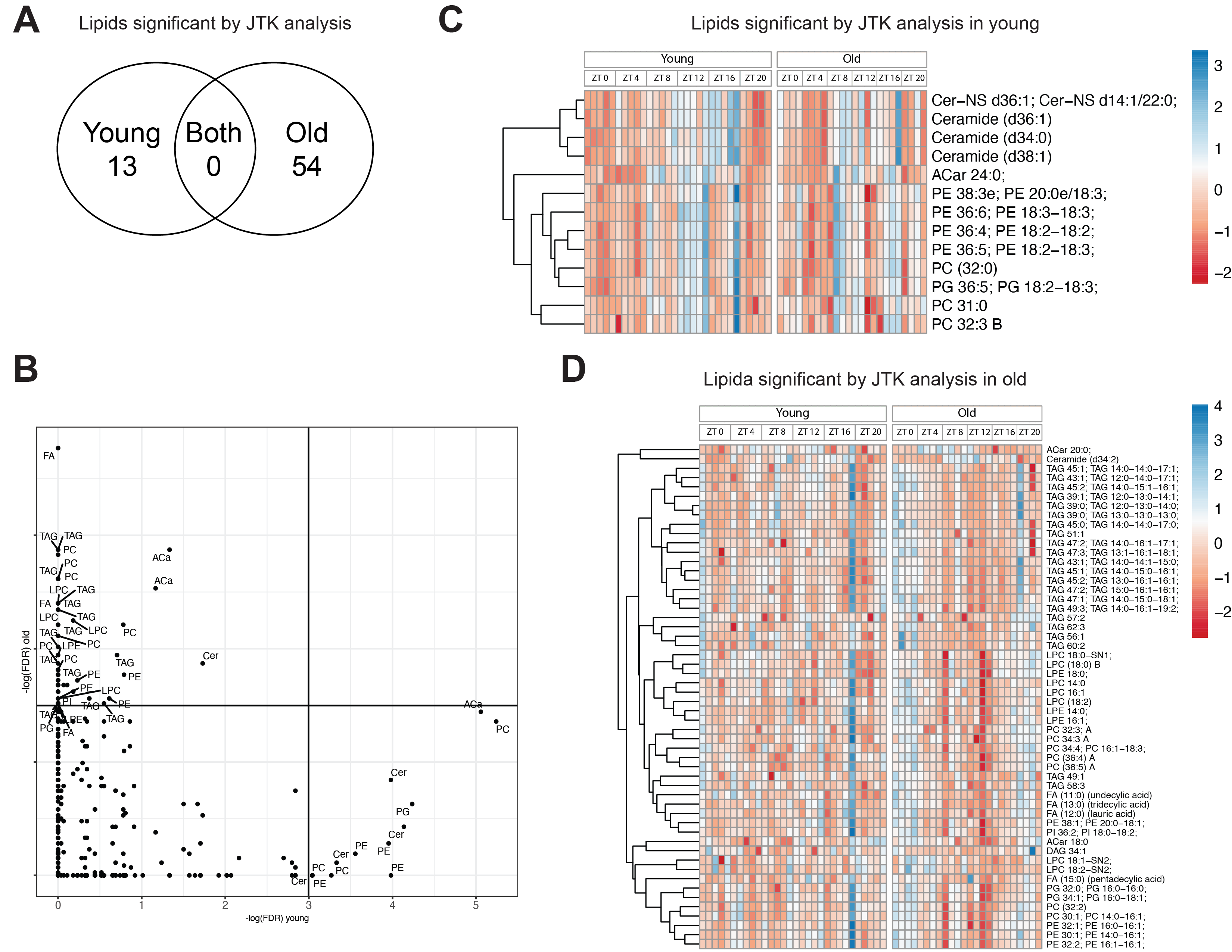
**

**Figure S1: Most of the lipids cycling in young and old fly heads are distinct.** Analysis of lipidomics data from both young and old flies at six circadian timepoints indicates that the lipids that showed significant daily variation by JTK analysis are distinct between groups (A). Note that triglycerides are strongly represented in the group of lipids that are distinct between groups. The data were analyzed using JTK analysis (BHQ<0.05), and are presented as a Venn diagram (A). An FDR plot provides visual representation of the significant cyclers in the young (clustered in the bottom right) and significant cyclers in old (clustered in the top right) (B). Heat maps of the data are shown to illustrate that more lipids are cycling in old flies (D) compared to young flies (C). For young N=5 and for old N=4 replicates per timepoint and each replicate is ~300 fly heads.

**Figure S2: Age does not affect feeding behavior in *Drosophila*.** There is no difference in the feeding pattern or quantity between young and old *Drosophila*. Average consumption in microliters per fly per hour for young and old white CantonS flies graphed per hour over 3 days (left). Error bars indicate SEM. Grayscale bars indicate light conditions. White: lights on (day); Black: lights off (night). Total 24-h food intake in microliters per day (Total 1=first 24-hr period) for each individual fly represented in the average line graph (right). There were no significant differences in the 24-h food intake between young and old flies. N=30-35 flies per group. Circles in the bar graph are individual fly data points, and summary statistics are displayed as mean +/- SEM. Means compared by Mann-Whitney test.


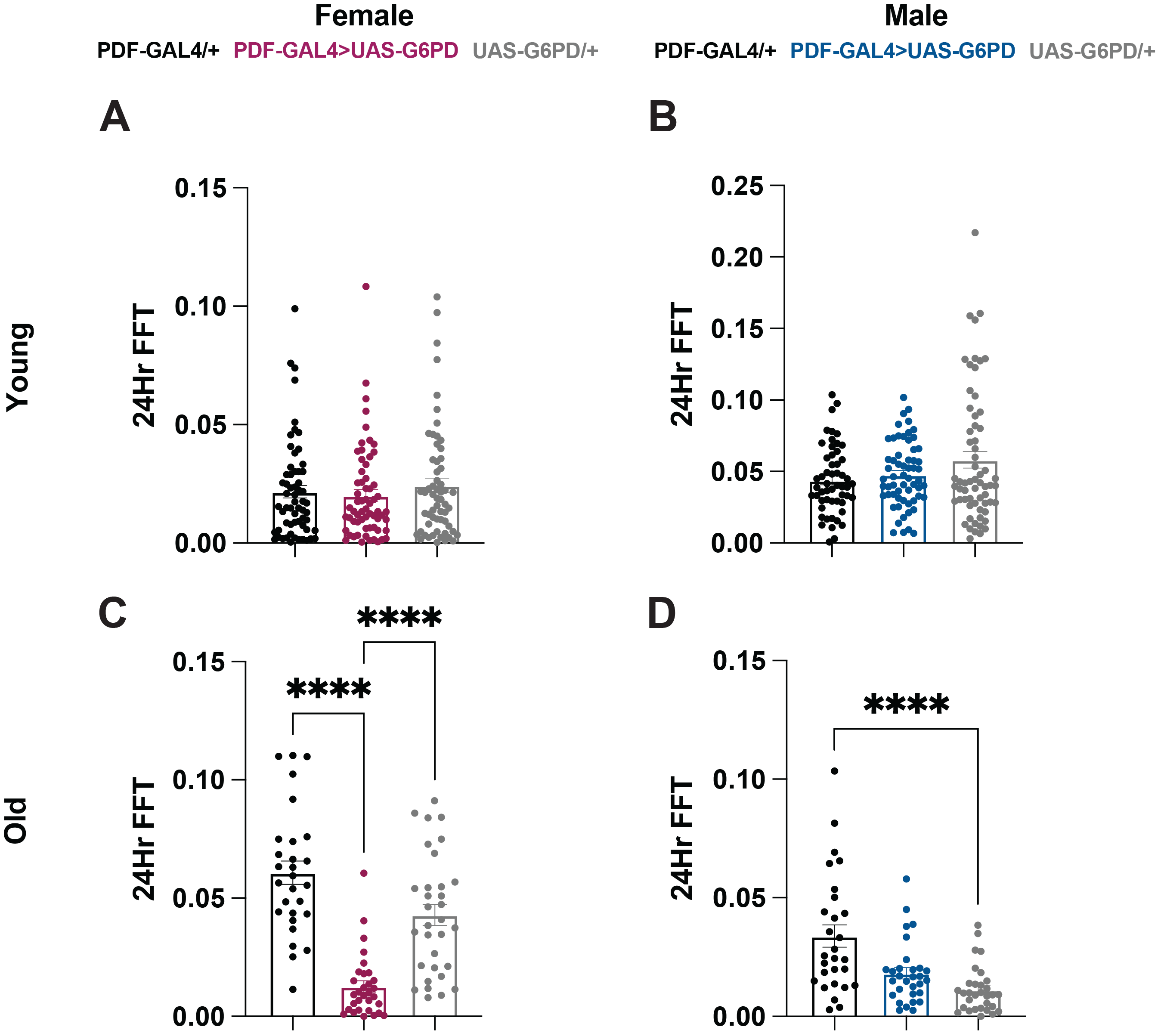


**Figure S3: G6PD Overexpression in PDF+ clock neurons only affects rhythmicity in old female flies.** 24hr FFT values, as a measure of circadian strength, in flies overexpressing G6PD in clock neurons. Locomotor activity was monitored for five days in DD. Only old female flies exhibited a decrease in rhythm strength (24hr FFT) when G6PD was overexpressed in PDF+ neurons (C). Circles are individual fly data points, and summary statistics are displayed as mean +/- SEM. Means compared by Kruskal-Wallis test followed by Dunn’s test (A-D). N=24-63 *****P* < 0.0001.


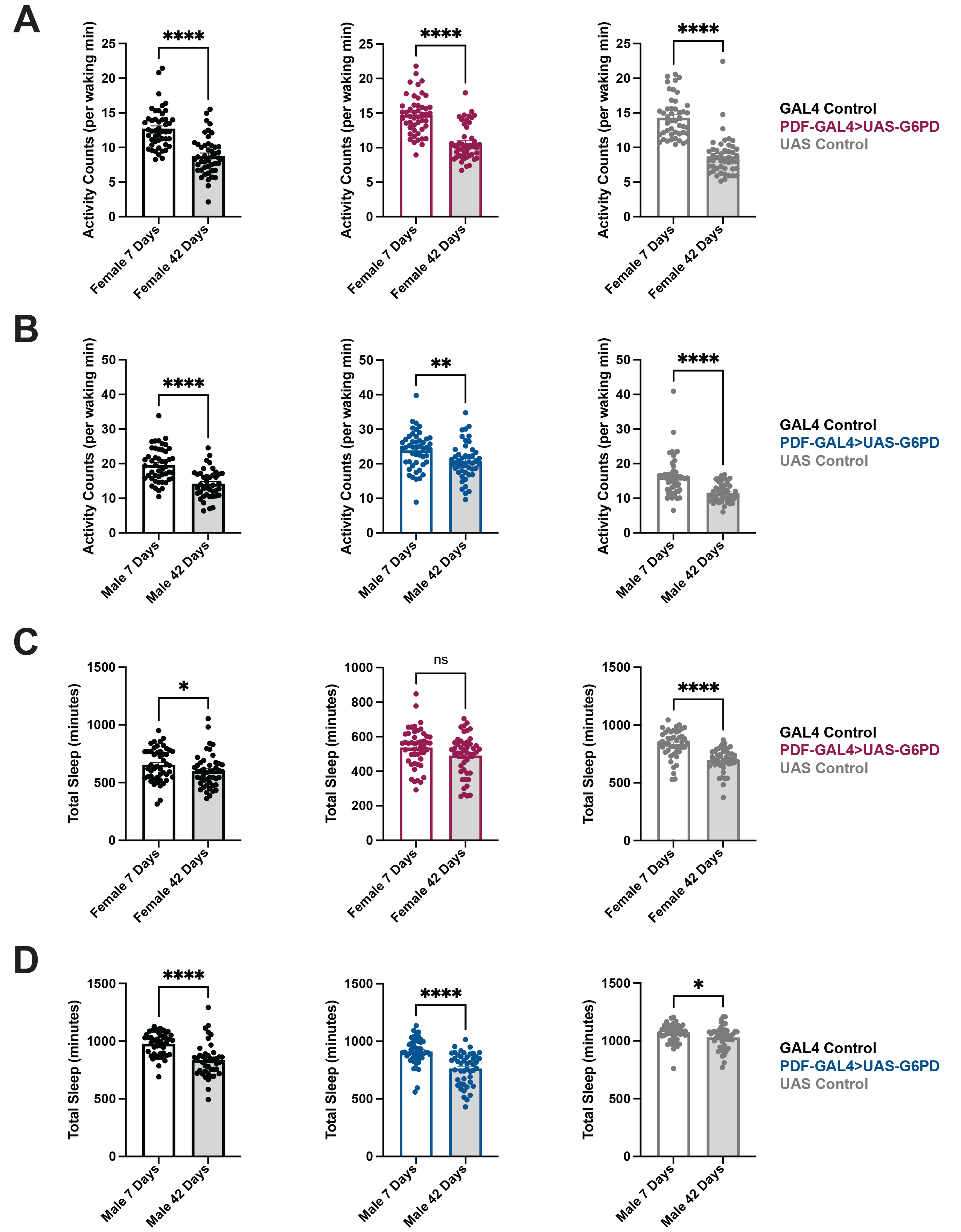


**Figure S4: Across** **genotypes,** **waking** **activity** **counts** **and** **total** **sleep** **decrease** **with** **age.** Between the ages of 7 days and 42 days waking activity counts decrease across all genotypes and both sexes (A,B). Additionally, as the flies age total sleep decreases (C, D) except for the PDF+ overexpression of G6PD female flies. Circles are individual fly data points, and summary statistics are displayed as mean +/- SEM. Means compared by Mann-Whitney (A-D) except for B left and middle (Welch t-test). N=41-48. **P* < 0.05, ***P* < 0.01, *****P* < 0.0001.


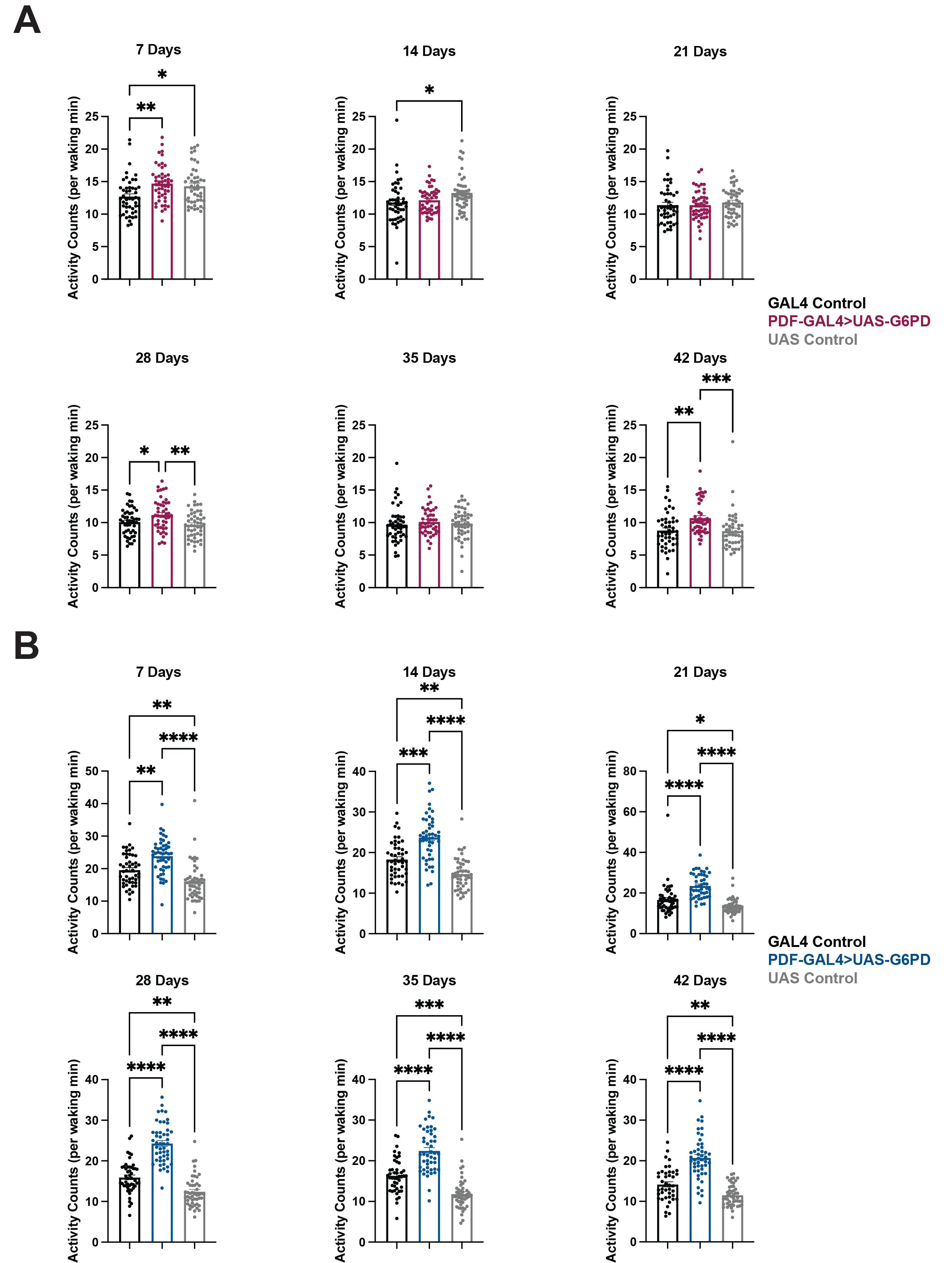
**Figure S5: G6PD overexpression in PDF+ clock neurons increases waking activity in male and old female flies.** Flies aged 7-42 days in multibeam monitors in 12:12 light:dark cycles were monitored for waking activity. At all ages investigated male flies with G6PD overexpression in PDF+ neurons exhibited increased waking activity compared to genetic controls. However, only 28 and 42 day old female flies exhibited significantly increased waking activity compared to controls. Circles are individual fly data points, and summary statistics are displayed as mean +/- SEM. Means are compared by one way ANOVA (A-Female) or Kruskal-Wallis (A-Male, B). N=41-48. **P* < 0.05, ***P* < 0.01, ****P* < 0.001, *****P* < 0.0001.


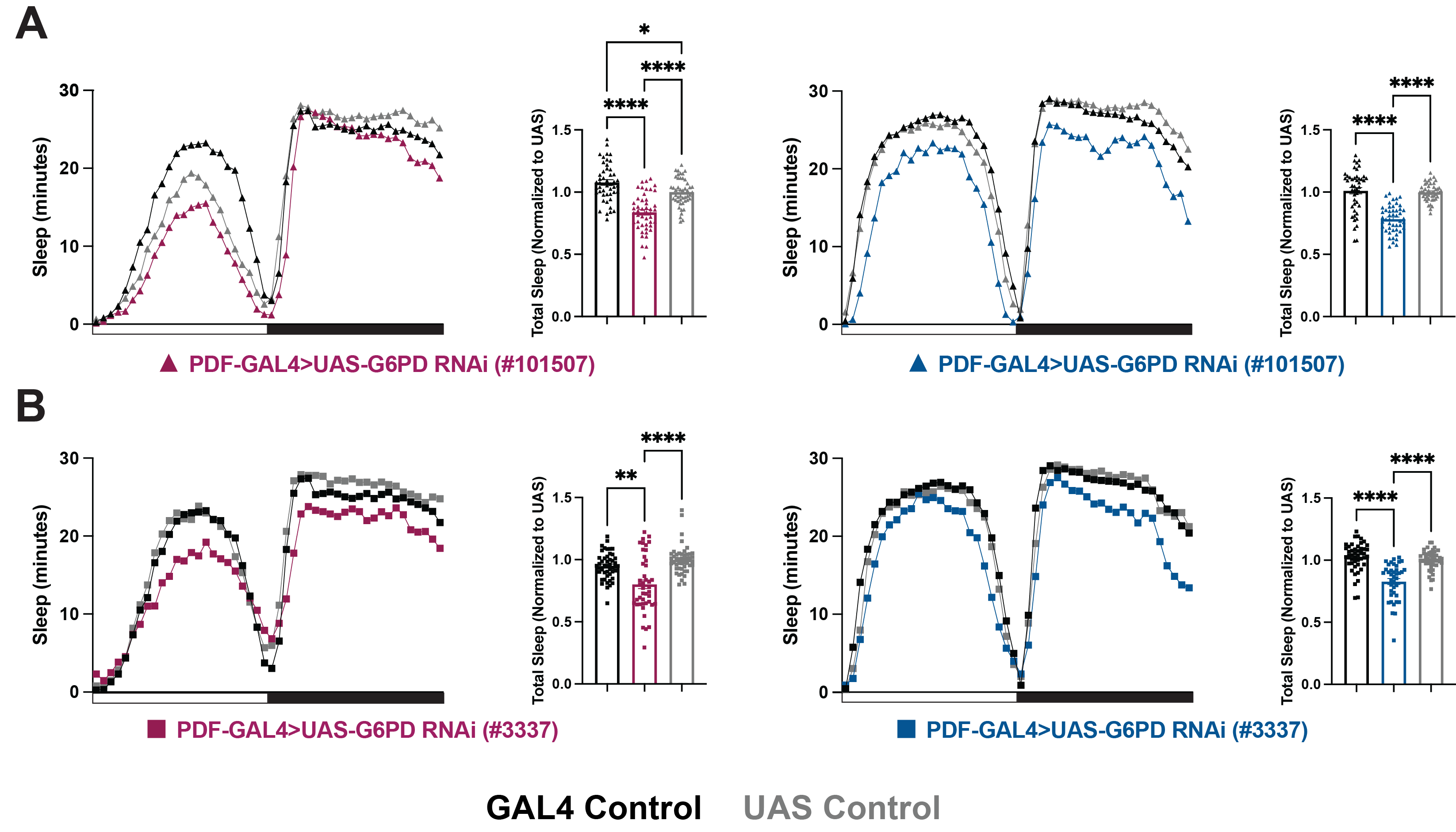


**Figure S6: G6PD Knockdown in PDF^+^ clock neurons decreases total sleep.** G6PD Knockdown in PDF^+^ clock neurons decreases night sleep (A-B). Average baseline sleep profiles in female (A, B left) and male (A,B right) transgenic young flies with the indicated genotypes are shown to illustrate effects on total sleep. White bar on X axis= lights on (day); Black bar on X axis= lights off (night). N=39-48 per group. The data for individual flies are presented with the mean +/- SEM for the group. Means are compared by one way ANOVA (A-Female) or Kruskal-Wallis (A-Male, B).**P* < 0.05, ***P* < 0.01, *****P* < 0.0001. N=38-48.
